# Void‐Assisted Extrinsic Charge Trapping Governs Hole Transport in Organic Semiconductors

**DOI:** 10.1002/smsc.70441

**Published:** 2026-11-10

**Authors:** Keigo Ishii, Kenichi Goushi, Youichi Tsuchiya, Kenji Ishida, Chihaya Adachi

**Affiliations:** ^1^ Center for Organic Photonics and Electronics Research (OPERA) Kyushu University Fukuoka Japan; ^2^ Department of Applied Quantum Physics & Nuclear Engineering Kyushu University Fukuoka Japan; ^3^ International Institute for Carbon Neutral Energy Research (I^2^CNER) Kyushu University Fukuoka Japan; ^4^ Quantum and Spacetime Research Institute (QSRI) Kyushu University Fukuoka Japan

**Keywords:** drift–diffusion model, host–guest system, molecular dynamics simulation, trap‐charge‐limited current

## Abstract

To realize electrically pumped organic lasing, single‐layer devices without organic heterointerfaces have been employed to suppress efficiency rolloff. In such architectures, on the other hand, extrinsic trap states result in trap‐assisted nonradiative recombination, thereby decreasing electroluminescence efficiency. Here, we elucidate the dominant factors governing extrinsic trap formation by combining current density (*J*)–voltage (*V*) characteristics of single‐layer hole‐only‐devices (HODs) with molecular dynamics (MD) simulations of 6,6′‐bibenzo[*d*]oxazole (BZ) derivatives exhibiting high photostability and low amplified spontaneous emission (ASE) thresholds. Drift‐diffusion analysis of the *J*–*V* characteristics reveals that 2,2′‐bis(4‐(9‐phenyl‐9*H*‐carbazol‐2‐yl)phenyl)‐6,6′‐bibenzo[*d*]oxazole (BZCz2) exhibits a higher density of extrinsic trap states than the other BZ derivatives studied. MD simulations further show that amorphous BZCz2 layers contain a high population of large voids arising from bulky substituents, potentially facilitating the incorporation of water clusters during vacuum deposition, thereby contributing to the formation of extrinsic hole traps. Furthermore, dispersing BZCz2 at low concentration in the host material of 4,4′‐bis(carbazol‐9‐yl)biphenyl (CBP), which exhibits dense molecular packing and minimal void formation, effectively suppresses extrinsic hole trapping in host–guest systems. These findings provide a strategy for suppressing extrinsic trap states and pave the way for highly efficient single‐layer EL devices.

## Graphical abstract

Molecular dynamics simulation reveals a higher population of large voids in BZCz2 than in BZCz, as shown by the cumulative void number density. Bulky substituents disrupt molecular packing and promote void formation, potentially facilitating water‐cluster incorporation during vacuum deposition and increasing extrinsic hole trapping. Molecular packing thus emerges as a key factor governing charge transport in organic semiconductors.

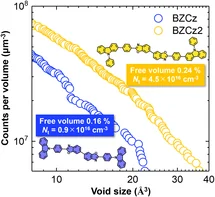

## 1 Introduction

Organic semiconductor materials offer several advantages, including lightweight and mechanically flexible devices, as well as virtually unlimited molecular design that enables precise control over optical and electronic properties, such as emission wavelength, energy levels, and charge‐transport characteristics. Owing to these advantages, organic materials have been widely employed in optoelectronic devices, as represented by organic light‐emitting diodes (OLEDs) and organic semiconductor lasers (OSLs) [1, 2, 3, 4, 5, 6, 7, 8, 9]. Particularly, in recent days, OSLs have attracted significant attention as next‐generation light‐emitting devices, with promising applications in XR displays, optical communications, and biosensing [4, 5, 6, 7, 8, 9]. Research on organic lasers has progressed steadily over the years and optically pumped solid‐state organic lasers have been extensively investigated [10, 11, 12, 13, 14, 15, 16]. The indication of electrically pumped lasing was demonstrated in 2019 [17]. More recently, lasing under indirect excitation by an OLED as an excitation source was reported in 2023 [18]. However, a persistent challenge for OSL devices is their extremely poor operational stability under extremely high excitation conditions. Achieving practical operation, therefore, requires a substantial reduction in the lasing threshold current density and the corresponding driving voltage.

Realizing electrically pumped lasing requires highly efficient electroluminescence (EL) under high current densities on the order of kA cm^−2^ to generate the high exciton densities necessary for population inversion [19, 20]. However, efficiency rolloff at such high current densities increases the threshold current density and degrades device performance. To maintain high EL efficiency under such conditions, a single‐layer device architecture has been developed because they suppress exciton–polaron annihilation induced by carrier accumulation at organic heterointerfaces [21, 22]. However, compared with multilayer devices, single‐layer devices generally exhibit lower maximum EL efficiencies due to the lack of efficient carrier confinement effect provided by heterostructures. Also, more fundamentally, one significant mechanism originates from nonradiative recombination via extrinsic trap states, which reduces EL efficiency through Shockley–Read–Hall (SRH) recombination between trapped and free charge carriers [23, 24, 25, 26]. This effect becomes especially critical in single‐layer devices with emissive layer thicknesses of approximately 200 nm, as typically assumed for OSL devices. It has been reported that, at an extrinsic trap density of 10^17^ cm^−3^, nonradiative SRH recombination can reduce EL efficiency by approximately 40% [25].

Here, we present a systematic investigation of extrinsic hole trapping in highly promising organic semiconductor laser materials, namely 6,6′‐bibenzo[*d*]oxazole (BZ) derivatives, which we previously reported to exhibit advanced photostability and low amplified spontaneous emission (ASE) thresholds [27]. We quantitatively evaluate the extrinsic trap density and trap depth by analyzing the current density (*J*)–voltage (*V*) characteristics of single‐layer hole‐only‐devices (HODs). Molecular dynamics (MD) simulations further suggest an association between void formation in amorphous films and extrinsic hole trapping. In particular, 2,2′‐bis(4‐(9‐phenyl‐9*H*‐carbazol‐2‐yl)phenyl)‐6,6′‐bibenzo[*d*]oxazole (BZCz2), which contains a large number of voids in the amorphous film, exhibits trap‐charge‐limited current (TCLC) behavior. Further, we demonstrate that dispersing BZCz2 at low concentration in a small‐void host material, 4,4′‐bis(carbazol‐9‐yl)biphenyl (CBP), effectively reduces the void population and suppresses extrinsic hole trapping. These results highlight the importance of tight molecular packing to suppress void formation and minimize extrinsic trapping in organic semiconductors and have implications for organic optoelectronic devices, including highly efficient single‐layer OLEDs [28, 29] and organic solar cells [30, 31].

## 2 Results and Discussion

### 2.1 Intrinsic and Extrinsic Trap States in Charge Transport

In the absence of charge‐carrier trapping, the current follows the Mott–Gurney square law for space‐charge‐limited currents (SCLC) and is proportional to the square of the applied voltage [32]. In contrast, when charge transport is limited by traps, the current exhibits a stronger voltage dependence and follows a power law $J\propto V^{m}$ with *m* > 2, characteristic of the TCLC behavior [33]. Charge‐carrier trapping can arise from both extrinsic and intrinsic sources. Extrinsic traps are typically associated with impurities such as water and oxygen, whereas intrinsic trapping originates from the amorphous nature of organic semiconductors. Possible intrinsic mechanisms include energetic disorder among hopping sites, variations in molecular packing within aggregates, and charge hopping opposite to the drift direction. These intrinsic effects act as transient traps that influence carrier mobility but do not form permanent trapping centers [34]. Therefore, when *J*–*V* characteristics exhibit TCLC behavior, the dominant trapping mechanism can be attributed primarily to extrinsic traps. Recent studies further indicate that hole trapping becomes significant when the highest occupied molecular orbital (HOMO) energy levels of organic materials are deeper than approximately −6.0 eV, whereas materials with shallower HOMO levels typically exhibit trap‐free hole transport [35].

### 2.2 *J–V* Characteristics of HODs Based on BZ Derivatives

Figure 1a−c  shows the *J*–*V* characteristics of the single‐layer HODs based on BZ derivatives: BZCz [2,2′‐bis(4‐(9*H*‐carbazol‐9‐yl)phenyl)‐6,6′‐bibenzo[*d*]oxazole], BZCz2 [2,2′‐bis(4‐(9‐phenyl‐9*H*‐carbazol‐2‐yl)phenyl)‐6,6′‐bibenzo[*d*]oxazole], and BZDP [4,4′‐([6,6′‐bibenzo[*d*]oxazole]‐2,2′‐diyl)bis(*N*,*N*‐diphenylaniline)]. The device architecture was designed to form ohmic contacts at the electrode–semiconductor interfaces [36], as described in the Experimental Section. The built‐in voltages (*V*_bi_) were estimated, following the reported methods (Figure S1) [37, 38]. The hole transport characteristics of BZCz and BZDP are consistent with the SCLC model incorporating the Poole–Frenkel effect, in which the carrier mobility depends on the electric field [39]. In contrast, BZCz2 exhibits TCLC behavior with a power law exponent of *m* = 3.6. At high voltages, the transition to a less steep *J*–*V* slope is observed, indicating that the current approaches the trap‐filled region.

**FIGURE 1:**
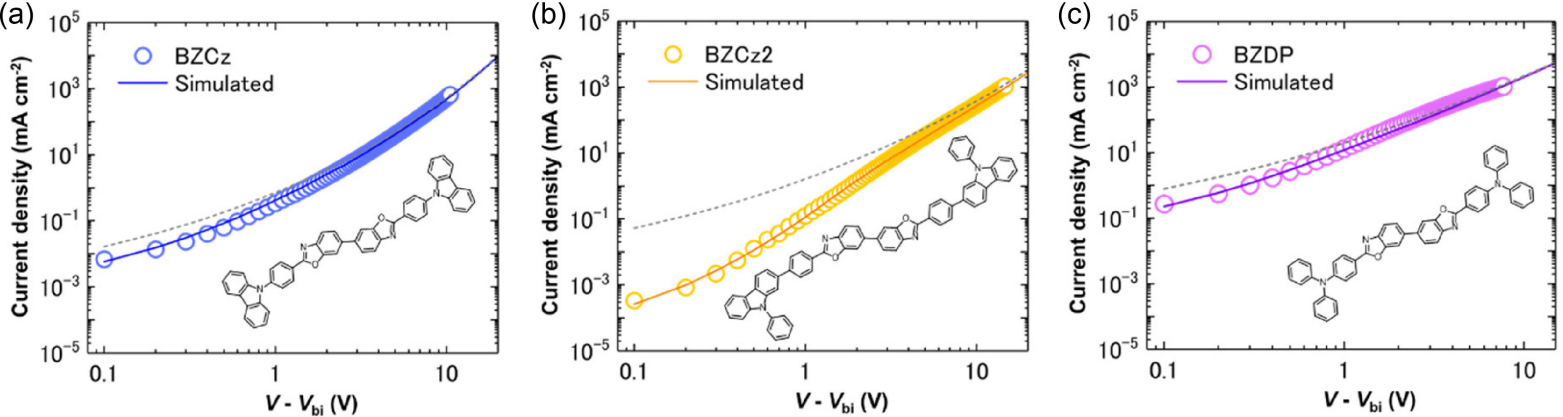
Experimental (symbols), simulated (solid lines), and trap‐free simulated (gray dashed lines) current density (*J*)–voltage (*V*) characteristics of HODs based on (a) BZCz (142 nm), (b) BZCz2 (145 nm), and (c) BZDP (100 nm). The inset shows the chemical structures of each molecule.

For BZDP, trap‐free hole transport is consistent with its HOMO energy level of −5.7 eV [27], which is shallower than the commonly reported trap‐free threshold of approximately −6.0 eV [35]. In contrast, BZCz and BZCz2 have HOMO energy levels of −6.1 and −5.9 eV, respectively [27], both located near this threshold. As discussed above, the TCLC observed in BZCz2 would not be attributed to intrinsic trapping but would be ascribed to extrinsic traps originating from factors such as water and oxygen incorporation.

Notably, all devices were fabricated by vacuum deposition and measured under a nitrogen‐filled glovebox (O_2_ < 0.1 ppm, H_2_O < 0.1 ppm) without air exposure. However, studies on residual impurities in vacuum chambers have shown that appreciable amounts of water and oxygen can be incorporated during film formation and they remain even under high‐vacuum conditions of 10^−5^ Pa [40]. Here, the collision frequency estimated from the partial pressure of water is approximately 5 × 10^12^ cm^−2^ s^−1^, comparable to the molecular collision frequency associated with film growth at a rate of 0.05 nm s^−1^ (10^12^–10^13^ cm^−2^ s^−1^). These considerations clearly suggest that water and oxygen can be incorporated into the organic film during vacuum deposition.

Water molecules can form water‐oxygen complexes, which exhibit solid‐state electron affinities of 2.9 and 3.7 eV [41]. Therefore, water molecules can act as electron traps; however, their deep gas‐phase ionization energy (12.65 eV) [42] prevents efficient hole trapping. In contrast, the situation changes markedly when water clusters are formed. When a water molecule is surrounded by a shell of H_2_O molecules, the surrounding dipoles stabilize the localized positive charge, resulting in a drastic reduction in the ionization energy to approximately 6 eV. Hole trapping induced by such water clusters has been supported by both experimental and theoretical studies [35, 43, 44], demonstrating that free volumes in amorphous organic films provide sufficient electrostatic stabilization. Therefore, water clusters are considered the most plausible origin of extrinsic hole traps in the present devices. Similarly, molecular oxygen is unlikely to act as a dominant hole trap because its gas‐phase ionization energy (12.06 eV) [45] is too deep relative to the HOMO levels of the investigated organic semiconductors.

Based on these considerations, the TCLC observed in BZCz2 can be attributed to extrinsic hole traps originating from water clusters. To quantitatively evaluate the extrinsic trap density and trap depth, a drift–diffusion model was applied to all *J*–*V* characteristics [43, 46]. This model self‐consistently solves the drift–diffusion equations together with the continuity equation and Poisson’s equation. The simulations were performed using the ATLAS device simulator. The trap distribution in organic semiconductor layers was described by a Gaussian density of states [47]. In addition, the carrier mobility was assumed to follow a Poole–Frenkel‐type electric‐field dependence [39]. As a result, the effects of intrinsic trapping are incorporated into the mobility, allowing the influence of extrinsic trapping to be clearly distinguished.

As shown by the solid lines in Figure 1, the simulated *J*–*V* characteristics accurately reproduce the experimental data for all devices. From these fits, the extracted trap density (*N*_t_) and trap depth (*E*_t_) are 0.9 × 10^16^ cm^−3^ and 0.25 eV for BZCz, 4.5 × 10^16^ cm^−3^ and 0.40 eV for BZCz2, and 1.8 × 10^16^ cm^−3^ and 0.30 eV for BZDP, respectively, demonstrating that BZCz2 possesses extrinsic traps that are both deeper and more abundant than those in others. The width of the Gaussian trap distribution (*σ*_t_), the zero‐field mobility (*μ*_0_), and the Poole–Frenkel factor (*β*) obtained from the *J*–*V* fitting are summarized in Table 1.

**TABLE 1:** Parameters obtained from drift–diffusion simulations by fitting the *J*–*V* characteristics of HODs.

Organic layer	Trap density, *N*_t_, cm^−3^	Trap depth, *E*t, eV	Width of Gaussian trap distribution, *σ*_t_, eV	Zero‐field mobility, *μ*_0_ a, cm^2^ V^−1^ s^−1^	Poole–Frenkel factor, *β* a, cm^1/2^ V^−1/2^
BZCz	0.9 × 10^16^	0.25	0.08	1.0 × 10^−6^	5.0 × 10^−3^
BZCz2	4.5 × 10^16^	0.40	0.10	3.0 × 10^−6^	2.6 × 10^−3^
BZDP	1.8 × 10^16^	0.30	0.10	2.3 × 10^−5^	1.0 × 10^−3^
CBP	1.3 × 10^16^	0.40	0.10	3.0 × 10^−6^	4.0 × 10^−3^
2 wt% BZCz: CBP	1.4 × 10^16^	0.40	0.10	4.0 × 10^−7^	4.7 × 10^−3^
2 wt% BZCz2: CBP	2.2 × 10^16^	0.40	0.10	7.0 × 10^−7^	4.0 × 10^−3^
2 wt% BZDP: CBP	1.8 × 10^16^	0.40	0.10	9.0 × 10^−7^	4.0 × 10^−3^
6 wt% BZCz: CBP	1.9 × 10^16^	0.40	0.12	2.5 × 10^−7^	4.3 × 10^−3^
6 wt% BZCz2: CBP	3.2 × 10^16^	0.40	0.10	6.0 × 10^−7^	4.3 × 10^−3^
6 wt% BZDP: CBP	1.8 × 10^16^	0.40	0.15	4.0 × 10^−7^	4.3 × 10^−3^
15 wt% BZCz: CBP	0.9 × 10^16^	0.25	0.15	5.0 × 10^−7^	5.0 × 10^−3^
15 wt% BZCz2: CBP	7.6 × 10^16^	0.40	0.10	2.5 × 10^−6^	4.3 × 10^−3^
15 wt% BZDP: CBP	4.5 × 10^16^	0.40	0.10	8.8 × 10^−7^	5.0 × 10^−3^
30 wt% BZCz: CBP	1.8 × 10^16^	0.25	0.10	5.0 × 10^−7^	5.7 × 10^−3^
30 wt% BZCz2: CBP	5.7 × 10^16^	0.50	0.10	3.0 × 10^−6^	2.6 × 10^−3^
30 wt% BZDP: CBP	2.2 × 10^16^	0.50	0.10	9.0 × 10^−7^	4.3 × 10^−3^

a The carrier mobility follows the Poole–Frenkel model and is expressed as *μ* = *μ*_0_ exp(*βE* ^1/2^), where *μ* is the carrier mobility, *μ*_0_ is the zero‐field mobility, *β* is the Poole–Frenkel factor, and *E* is the electric field [39].

To clarify how the trap density and trap depth influence the *J*–*V* characteristics, simulations were performed in which the trap depth was varied at a fixed trap density, and, conversely, the trap density was varied at a fixed trap depth. The corresponding simulated *J*–*V* characteristics are shown in Figure S2. When the trap density is low (1.0 × 10^16^ cm^−3^), increasing the trap depth has little effect on the *J*–*V* characteristics. In contrast, at higher trap densities, the deeper traps lead to a pronounced reduction in the current density at low voltages and produce a steeper slope in the trap‐limited region.

Since the HOMO energy levels of BZCz and BZCz2 are close to the boundary of the trap‐free window and differ only in their terminal donor groups, the origin of the pronounced difference in extrinsic trap density cannot be explained solely by energetic considerations. Note that BZCz exhibits a HOMO level of −6.1 eV, whereas BZCz2 has a bit shallower HOMO level of −5.9 eV. From an energetic standpoint, BZCz2 would therefore be expected to be less susceptible to trap effects than BZCz. However, the experimentally observed behavior is inconsistent with this expectation, indicating that factors other than energy levels influence the formation of extrinsic traps.

### 2.3 MD Simulation of BZCz and BZCz2

A comparison between the molecular structures of BZCz and BZCz2 reveals that BZCz2 contains bulky 9‐phenyl‐9*H*‐carbazol‐2‐yl substituents, which can hinder molecular packing (Figure 2a). Based on this structural difference, we focus on MD simulations to elucidate the role of molecular packing and free volume (Figure 2b,c). The MD simulations of the amorphous films were performed as described in the Experimental Section. The system density, potential energy, and simulation‐cell volume reached stable values during the final equilibration stage, indicating that the systems had reached stable states within the simulation timescale (Figures 2d and S3). To evaluate the free‐volume fraction, additional simulations were performed using the same simulation protocol with different numbers of molecules, and the resulting free‐volume fractions were averaged (Figure S4). The average free‐volume fraction is higher for BZCz2 (0.24%) than for BZCz (0.16%) (Figure 2e). Furthermore, the cumulative void distributions shown in (Figure 2f) clearly demonstrate that BZCz2 contains a greater population of large voids. Although the bulky substituents in BZCz2 are likely to influence its packing behavior, the larger free volume should not be attributed solely to steric bulk. Instead, it likely arises from the combined effects of steric bulk, molecular shape, conformational flexibility, and intermolecular packing. This increased population suggests that void formation is more likely in BZCz2 during vacuum deposition, thereby facilitating the incorporation of water molecules as clusters. Such void‐assisted incorporation of water clusters provides a plausible explanation for the higher extrinsic trap density in BZCz2.

**FIGURE 2:**
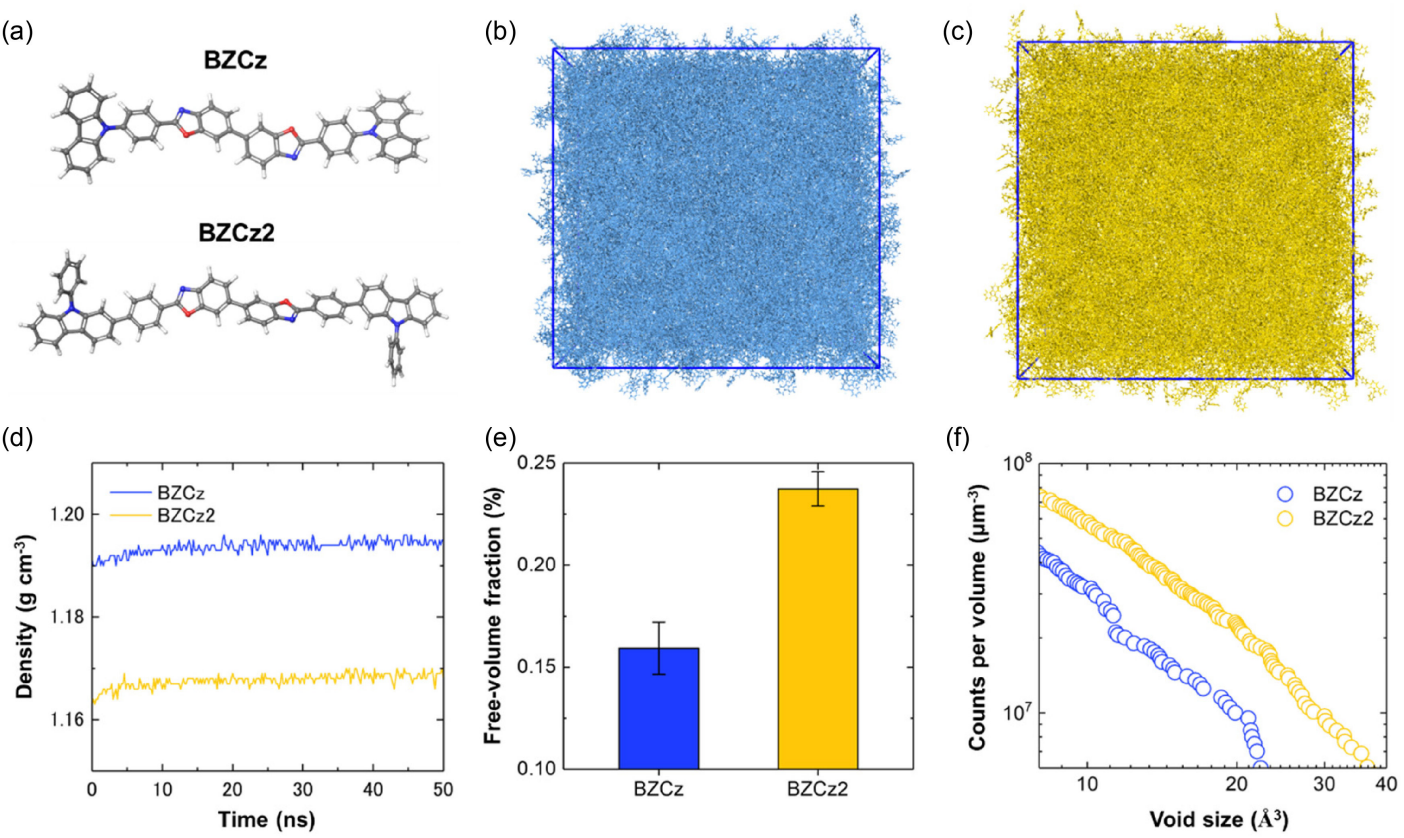
(a) DFT‐optimized molecular structures of BZCz and BZCz2. Molecular dynamics (MD)‐simulated amorphous film structure of (b) BZCz and (c) BZCz2 containing 2000 molecules. (d) Time evolution of the system density during the final equilibration stage for the 2000‐molecule systems. (e) Average free‐volume fractions calculated from systems with different numbers of molecules using the same simulation protocol. Error bars represent the standard deviation. (f) Cumulative void number density as a function of void size for the 2000‐molecule systems of BZCz and BZCz2.

### 2.4 Hole Transport and Molecular Packing in CBP

As described above, the larger void size and higher void population observed in the amorphous BZCz2 film are expected to promote the incorporation of water clusters, leading to extrinsic hole trapping. To further elucidate the relationship between molecular packing and extrinsic trap formation, we examined 4,4′‐bis(carbazol‐9‐yl)biphenyl (CBP), a compact molecule widely used as a host material in OLEDs and organic lasers (Figure 3a). As shown in Figure S4, the amorphous CBP film exhibits a markedly low free‐volume fraction of 0.13%. Moreover, the cumulative void‐size distribution (Figure 3b) shows that the population of large voids is lower in CBP than in BZCz2, indicating highly efficient molecular packing. These results suggest that compact molecular structures lacking bulky substituents, such as CBP, intrinsically suppress void formation in the amorphous films.

**FIGURE 3:**
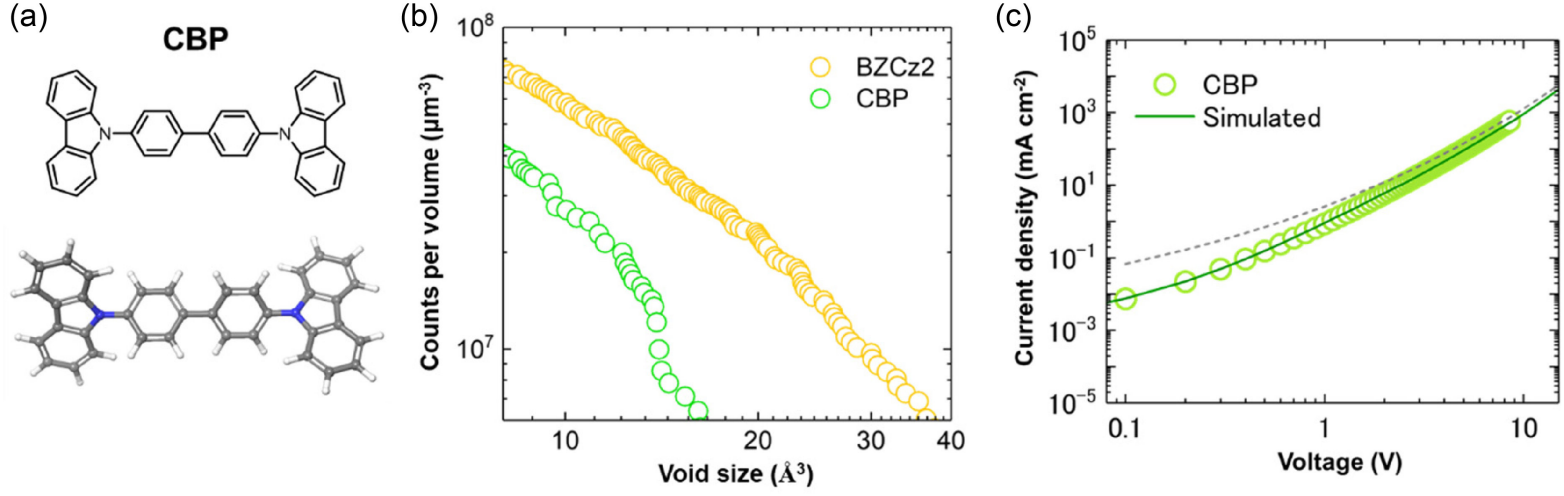
(a) Chemical structure and DFT‐optimized molecular structure of CBP. (b) Cumulative void number density as a function of void size for the 2000‐molecule systems of BZCz2 and CBP. (c) Experimental (symbols), simulated (solid lines), and trap‐free simulated (gray dashed lines) *J*–*V* characteristics of a CBP (126 nm) HOD.

These structural characteristics are directly reflected in charge‐transport properties. As shown in (Figure 3c), the experimentally extracted extrinsic trap density in a CBP film is as low as 1.3 × 10^16^ cm^−3^ (the built‐in voltage was set to 0 V, as shown in Figure S5). Although the HOMO level of CBP (−6.0 eV) lies at the boundary of the trap‐free window [35], the small free volume effectively suppresses the incorporation of water clusters. These results suggest that controlling void formation through molecular packing is an important factor in mitigating extrinsic charge‐carrier trapping, complementing energy‐level considerations in organic semiconductor materials [35].

### 2.5 Host–Guest System

Given the small size and low population of voids in the CBP film, dispersing BZCz2 into a CBP host is expected to effectively mitigate extrinsic hole trapping associated with BZCz2. In general, host–guest systems, in which emissive molecules are diluted within a host matrix, are known to suppress molecular aggregation and concentration quenching at high guest concentrations, thereby enabling high photoluminescence efficiency. At the same time, charge trapping originating from guest molecules must be carefully considered in such systems. MD‐based density‐of‐states (DOS) calculations show that the ionization energy of BZCz2 lies well within the distribution of ionization energies of CBP (Figure S6). This energetic overlap indicates that BZCz2 does not form a pronounced energetic trap relative to the CBP host, allowing holes localized at BZCz2 sites to readily escape.

Figure 4a–d show the *J*–*V* characteristics of BZCz2‐doped CBP HODs at various doping concentrations (2, 6, 15, and 30 wt%). At low BZCz2 concentrations of 2 and 6 wt%, the hole transport behavior closely resembles that of the neat CBP film. This observation indicates that dispersing BZCz2 at low concentration within a CBP host with small voids effectively suppresses extrinsic traps associated with BZCz2. In contrast, at higher doping concentrations of 15 and 30 wt%, the *J*–*V* characteristics change markedly, exhibiting trap‐limited hole transport at low voltages. As summarized in (Figure 4e), these results indicate that the influence of BZCz2‐induced extrinsic traps becomes dominant above the critical BZCz2 concentration.

**FIGURE 4:**
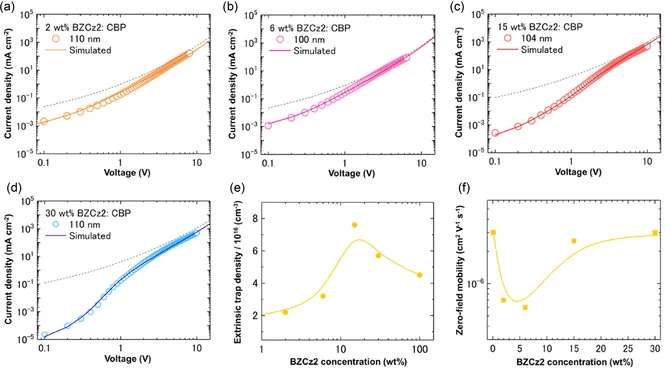
Experimental (symbols), simulated (solid lines), and trap‐free simulated (gray dashed lines) *J*–*V* characteristics of BZCz2 HODs with BZCz2 concentrations of (a) 2 wt%, (b) 6 wt%, (c) 15 wt%, and (d) 30 wt% in CBP host. (e) Extracted extrinsic trap density and (f) zero‐field hole mobility as functions of BZCz2 doping concentration.

In addition, guest‐induced traps in host–guest systems have been reported to intrinsically affect carrier mobility [48]. Consistent with this behavior, (Figure 4f) shows that zero‐field carrier mobilities in BZCz2:CBP codeposited films decrease by approximately one order of magnitude even at low doping concentrations. This reduction in mobility suggests that guest‐induced traps act primarily as transient trap sites, i.e., energetic disorder among hopping sites, variations in molecular packing between host and guest molecules, modulating charge transport without forming permanent traps.

Figure 5a–d show the *J*–*V* characteristics of HODs based on host–guest systems with BZCz or BZDP as guest molecules at doping concentrations of 15 and 30 wt%. The corresponding *J*–*V* characteristics at lower concentrations (2 and 6 wt%) are presented in Figure S7. From these results, BZCz, which exhibits trap‐free hole transport and a relatively low void population, maintains a similarly low trap density across all investigated doping concentrations (Figure 5e), comparable to those of neat CBP and BZCz films. This behavior indicates that co‐deposition of molecules with a low void population preserves trap‐free hole transport.

**FIGURE 5:**
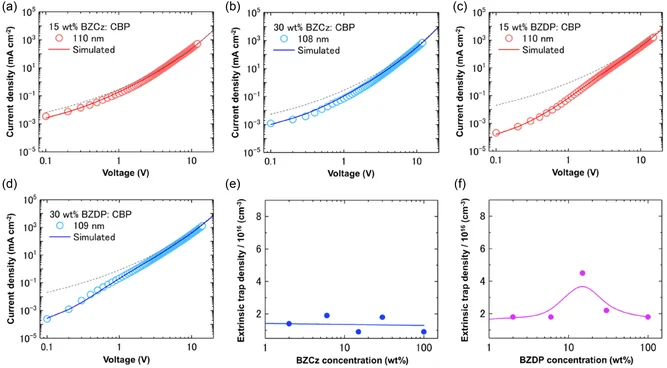
Experimental (symbols), simulated (solid lines), and trap‐free simulated (gray dashed lines) *J*–*V* characteristics of host–guest HODs: (a) 15 wt% BZCz, (b) 30 wt% BZCz, (c) 15 wt% BZDP, and (d) 30 wt% BZDP in the CBP host. Extracted extrinsic trap density as a function of doping concentration for (e) BZCz and (f) BZDP, respectively.

Although BZDP‐doped host–guest systems exhibit low trap densities at the low and high concentrations, the trap density increased at around 15 wt% and was estimated to be 4.5 × 10^16^ cm^−3^ (Figure 5f). The HOD, based on neat BZDP, shows trap‐free hole transport (Figure 1c). The guest‐induced trapping may contribute to the shallow HOMO level of BZDP. To evaluate this possibility, we calculated the molecular density at 15 wt%. The calculated molecular density exceeds 10^19^ cm^−3^, which is significantly higher than the estimated trap density. This comparison suggests that the dominant trapping mechanism originates from extrinsic traps.

To elucidate the origin of this behavior, MD simulations of the host–guest systems at 6 and 15 wt% were performed. For comparison, the MD simulation of a neat amorphous BZDP film was also conducted. As shown in Figure S8, the amorphous BZDP film exhibits a relatively high population of large voids. The relatively high population of large voids could provide more sites capable of accommodating water clusters. Nevertheless, the shallow HOMO level of BZDP enables trap‐free hole transport in the neat film. The MD simulation results for BZDP:CBP blend films are shown in Figure S9a. In the host–guest systems, the population of large voids at 6 wt% remains low and is comparable to that in the neat CBP film, consistent with the low extrinsic trap density observed experimentally. In contrast, a higher population of large voids is observed at 15 wt%, where the extrinsic trap density also increases. At sufficiently high BZDP concentrations, hopping between BZDP molecules would dominate carrier transport. Consequently, the trap density decreases in this high‐concentration region.

For BZCz2, a pronounced increase in trap density is also observed around 15 wt%, as shown in (Figure 4e). According to MD simulations (Figure S9b), the population of large voids at 6 wt% remains comparable to that in the neat CBP film, whereas a higher population of large voids is observed at 15 wt%. A similar increase is observed experimentally in the extrinsic trap density at this concentration.

In the MD simulations, the overall free‐volume fraction at 15 wt% remains relatively small (~0.14%) and is lower than that of the corresponding neat films in all host–guest systems. However, guest molecules may not be distributed as ideally or as uniformly in real films as assumed in the simulations. Local aggregation of guest molecules and spatial inhomogeneity in doped films could therefore influence void formation, and the contribution of void‐assisted extrinsic trapping in real films may differ from that predicted by the MD simulations.

## 3 Conclusion

In this study, we elucidated the correlation between hole‐transport characteristics in BZ derivatives and the presence of voids in amorphous films using a combination of experimental and simulation approaches. BZCz2 exhibits a larger population of large voids, which is likely associated with differences in molecular structure and packing, including steric bulk, molecular shape, and conformational flexibility. These large voids can provide sites capable of accommodating water clusters during film formation, potentially contributing to the higher extrinsic hole‐trap density observed for BZCz2. We further demonstrate that dispersing BZCz2 at low concentrations within a compact CBP host with small voids effectively mitigates the impact of extrinsic traps. In contrast, at higher doping concentrations, local molecular aggregation and structural inhomogeneity can contribute to increased void formation, accompanied by more pronounced trap‐limited hole transport. These results demonstrate that extrinsic charge‐carrier trapping in organic semiconductors can be associated not only with energy levels but also with void formation in amorphous films. This insight provides a strategy for mitigating extrinsic trap states and paves the way for highly efficient single‐layer OLEDs. In our subsequent study, we would like to clarify the electron trapping effect with electron‐only devices and obtain a comprehensive understanding of bipolar devices.

## 4 Experimental Section

### 4.1 Materials

BZ derivatives were synthesized according to previously reported methods [27]. All materials were purified by vacuum train sublimation. Other materials were purchased commercially.

### 4.2 Device Fabrication

Single‐layer HODs were fabricated on indium tin oxide (ITO)‐coated glass substrates by vacuum deposition under a pressure of below 4.0 × 10^−4^ Pa, without exposure to the atmosphere. The device structure is ITO (100 nm)/MoO_3_ (7 nm)/4CzIPN (4 nm)/organic layer/MoO_3_ (7 nm)/Al (70 nm), where 4CzIPN is 1,2,3,5‐tetrakis(carbazol‐9‐yl)‐4,6‐dicyanobenzene. An interlayer with a higher ionization energy than the semiconductor promotes Ohmic contact formation at high‐work‐function metal–oxide electrodes by electrostatically decoupling the interface and realigning the Fermi level [36]. MoO_3_, 4CzIPN, and organic layers were deposited through a metal mask at a deposition rate of 0.01, 0.01, and 0.05 nm s^−1^, respectively. After deposition, the metal mask was replaced with another for cathode deposition inside a nitrogen‐filled glovebox. Aluminum electrodes were deposited on the organic layers at rates of 0.02–0.2 nm s^−1^.

### 4.3 *J*–*V* Measurement

The *J*–*V* measurements for fabricated devices were performed using a semiconductor parameter analyzer (B1500A, Keysight) within a nitrogen‐filled glove box (O_2_ < 0.1 ppm, H_2_O < 0.1 ppm). The devices were measured without air exposure.

### 4.4 Device Simulation

Device simulations were performed using the ATLAS module of Silvaco, employing a 2D steady‐state drift–diffusion model self‐consistently coupled with the continuity equation and Poisson’s equation. Carrier densities were described using a parabolic density of states under Maxwell–Boltzmann statistics. Trap states were modeled with a Gaussian distribution. Electric‐field dependent mobilities followed a Poole–Frenkel model.

### 4.5 Molecular Dynamics Simulation

The MD simulations were performed as follows. Isolated molecules were first optimized using density functional theory (DFT) with the B3LYP functional and 6‐31G** basis set in the gas phase by using Jaguar (Schrödinger, Inc.). The DFT‐optimized structures were subsequently used to construct amorphous systems using the Disordered System Builder implemented in the Materials Science Suite 2026–1 (Schrödinger, Inc.). The molecules were randomly packed into periodic simulation cells with an initial density of 0.5 g cm^−3^. All‐atom MD simulations were carried out using the Desmond simulation engine with the OPLS4 force field [49], in which bonded and nonbonded parameters as well as atomic partial charges were automatically assigned. The applicability of OPLS4 to the present molecular systems was assessed by comparing torsional potential‐energy profiles for representative rotatable bonds of BZCz, BZCz2, and BZDP with those obtained from DFT calculations at the B3LYP/6‐31G** level. OPLS4 reproduced the principal energy minima and overall torsional profiles, with deviations comparable to those obtained for CBP as a reference system [50] (Figure S10). Periodic boundary conditions were applied in all three dimensions. Electrostatic interactions were treated using the particle mesh Ewald (PME) method, while short‐range nonbonded interactions were evaluated using a cutoff distance of 9 Å. The equations of motion were integrated using the reversible reference system propagator algorithm (RESPA) integrator with a time step of 2 fs. Temperature was controlled using the Nosé–Hoover thermostat. In the NPT simulations, pressure was maintained at 1 atm using the Martyna–Tobias–Klein barostat with isotropic pressure coupling. Prior to the production simulations, the initially generated amorphous structures were relaxed using the Materials Relaxation protocol implemented in the Materials Science Suite. The protocol consisted of 20 ps of Brownian dynamics in the canonical (NVT) ensemble at 10 K, followed by 20 ps of Brownian dynamics in the isothermal–isobaric (NPT) ensemble at 100 K and 100 ps of NPT molecular dynamics equilibration at 300 K. This stepwise relaxation procedure was used to relax unfavorable intermolecular contacts and high‐energy local configurations in the initially generated amorphous structures. Subsequently, production simulations were performed in the NPT ensemble at 300 K and 1 atm for 10 ns. The systems were then heated from 300 K to 800 K at a rate of 0.5 K ps^−1^, followed by equilibration in the NPT ensemble at 800 K and 1 atm for 10 ns. Subsequently, the systems were cooled from 800 to 300 K at a cooling rate of 0.5 K ps^−1^. Finally, production simulations were performed in the NPT ensemble at 300 K and 1 atm for 50 ns. Equilibration of the amorphous structures was verified by monitoring the convergence of the system density, potential energy, and simulation cell volume (Figures 2d and S3). Free‐volume analysis was performed using the Free Volume Analysis module implemented in the Materials Science Suite 2026–1. The simulation cell was represented on a 3D grid with a spacing of 0.5 Å, and a probe radius of 1.4 Å was used to identify probe‐accessible free‐volume regions. Individual connected free‐volume regions were identified as separate voids, and the volume of each void was calculated from the corresponding grid points. The free‐volume fraction was calculated as the ratio of the total free volume to the simulation‐cell volume. Because the simulation‐cell volumes differed among the investigated systems, the cumulative void‐size distribution was subsequently normalized by the corresponding simulation‐cell volume. Specifically, the cumulative void number density at a given void volume *V* was defined as the number of voids having volumes equal to or larger than *V*, divided by the simulation‐cell volume. Individual voids were approximated as volume‐equivalent spheres.

## Funding

This work was supported by JSPS KAKENHI Grant Numbers JP23H05406 and 23K20039.

## Conflicts of Interest

The authors declare no conflicts of interest.

## Supporting information

Supplementary Material

## Data Availability

The data that support the findings of this study are available from the corresponding author upon reasonable request.
