# Supplementary material for: Void‐Assisted Extrinsic Charge Trapping Governs Hole Transport in Organic Semiconductors
Source: Small Sci. 2026 Nov 10;6(11):e70441. doi: 10.1002/smsc.70441 (PMC13749069; doi:10.1002/smsc.70441)
Supplement: Supplementary file 1 [file SMSC-6-e70441-s001.pdf]

Supporting Information

Void-Assisted Extrinsic Charge Trapping Governs Hole Transport in Organic Semiconductors

Keigo Ishii, Kenichi Goushi, Youichi Tsuchiya, Kenji Ishida, and Chihaya Adachi**

K. Ishii, Y. Tsuchiya, C. Adachi
Center for Organic Photonics and Electronics Research (OPERA), Kyushu University, 744 Motooka, Nishi, Fukuoka 819-0395, Japan
E-mail: adachi@opera.kyushu-u.ac.jp

K. Goushi, K. Ishida
Department of Applied Quantum Physics & Nuclear Engineering, Kyushu University, 744 Motooka, Nishi, Fukuoka 819-0395, Japan
E-mail: goshi.kenichi.540@m.kyushu-u.ac.jp

C. Adachi
International Institute for Carbon Neutral Energy Research (I2CNER), Kyushu University, 744 Motooka, Nishi, Fukuoka 819-0395, Japan

C. Adachi
Quantum and Spacetime Research Institute (QSRI), Kyushu University, Nishi, Fukuoka 819-0395, Japan

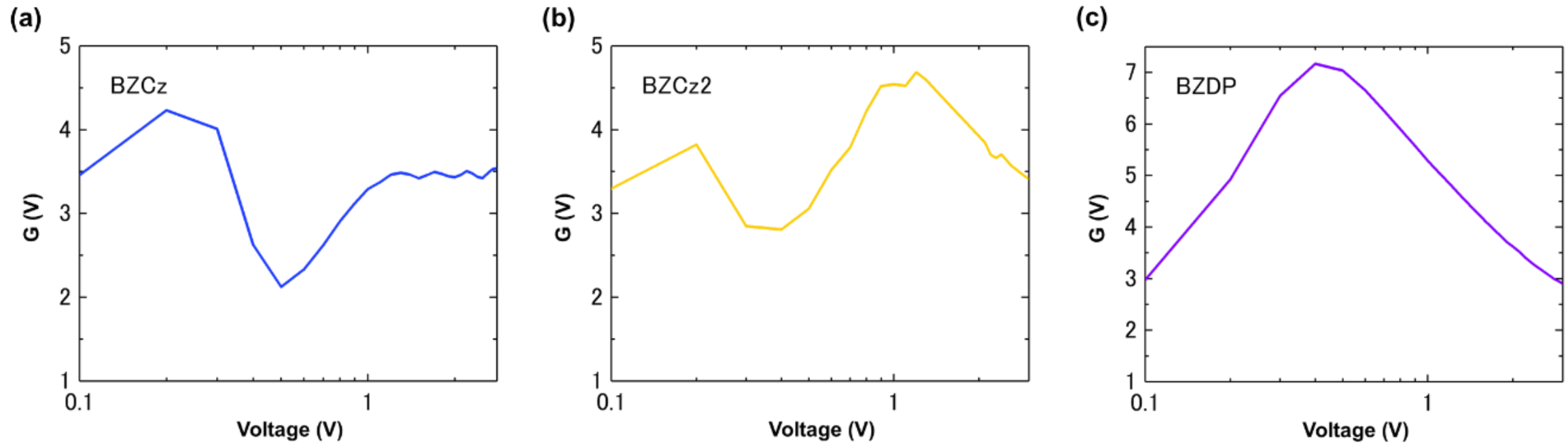

Figure S1. G(V)–V characteristics of HODs based on (a) BZCz, (b) BZCz2, and (c) BZDP. The function G(V) is expressed as $G(\mathrm{V}) = \mathrm{dlog}(J) / \mathrm{dlog}(V)$. The peak voltage V_α of G(V) is linearly related to the built-in voltage V_{bi} and is described as $V_\alpha = 0.925V_{\mathrm{bi}} - 0.25$.[1] Based on this equation, the built-in voltages V_{bi} were set to 0.5 V at BZCz, 0.5 V at BZCz2, and 0.7 V at BZDP.

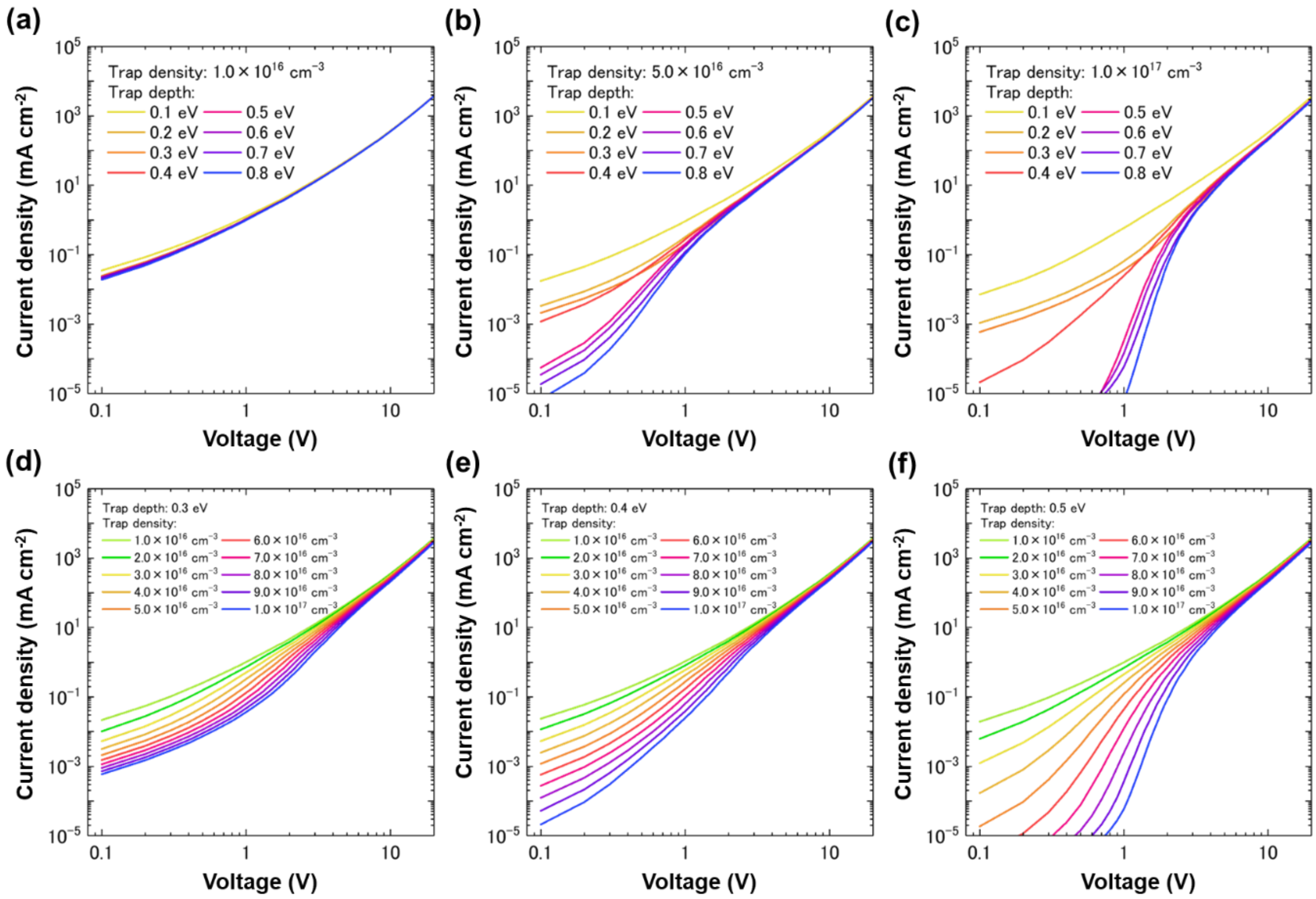

Figure S2. Simulated J–V characteristics for an organic layer thickness of d = 100 nm. (a–c) Simulations with fixed trap density N_t = 1.0×10^{16} cm^{-3}, 5.0×10^{16} cm^{-3}, and 1.0×10^{17} cm^{-3}, respectively, and varying trap depths E_t. (d–f) Simulations with fixed trap depths E_t = 0.30 eV, 0.40 eV, and 0.50 eV, respectively, and varying trap densities N_t. The width of the Gaussian trap distribution was set to σ_t = 0.10 eV in all simulations.

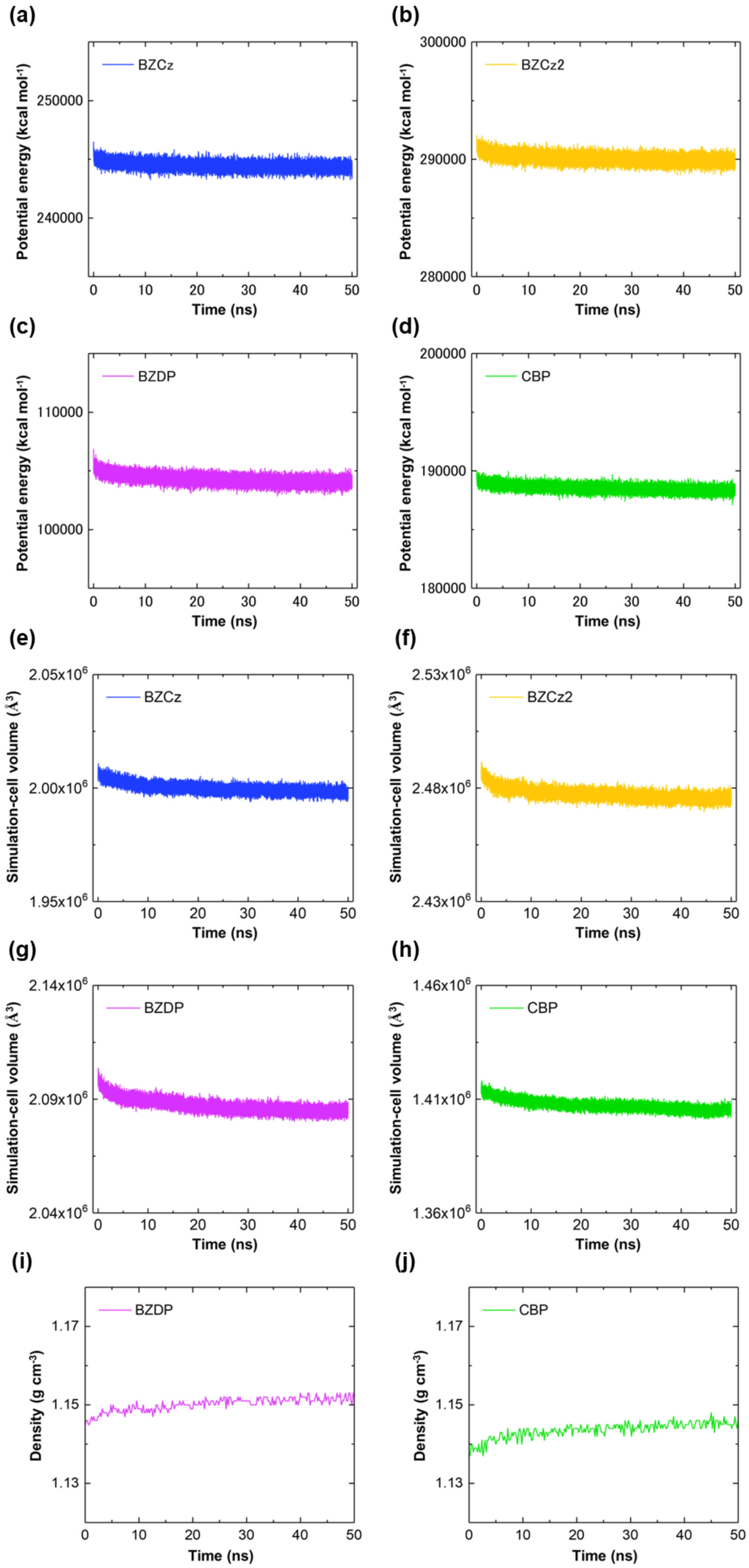
(a)
BZCz
Potential energy (kcal mol-1)
250000
240000
0
10
20
30
40
50
Time (ns)
(b)
BZCz2
Potential energy (kcal mol-1)
300000
290000
280000
Time (ns)
(c)
BZDP
Potential energy (kcal mol-1)
110000
100000
Time (ns)
(d)
CBP
Potential energy (kcal mol-1)
200000
190000
180000
Time (ns)
(e)
BZCz
Simulation-cell volume (Å3)
2.05x106
2.00x106
1.95x106
Time (ns)
(f)
BZCz2
Simulation-cell volume (Å3)
2.53x106
2.48x106
2.43x106
Time (ns)
(g)
BZDP
Simulation-cell volume (Å3)
2.14x106
2.09x106
2.04x106
Time (ns)
(h)
CBP
Simulation-cell volume (Å3)
1.46x106
1.41x106
1.36x106
Time (ns)
(i)
BZDP
Density (g cm-3)
1.17
1.15
1.13
Time (ns)
(j)
CBP
Density (g cm-3)
1.17
1.15
1.13
Time (ns)

Figure S3. Time evolution of the potential energy for (a) BZCz, (b) BZCz2, (c) BZDP, and (d) CBP; simulation-cell volume for (e) BZCz, (f) BZCz2, (g) BZDP, and (h) CBP; and system density for (i) BZDP and (j) CBP during the final 50 ns NPT equilibration at 300 K and 1 atm. All systems consisted of 2000 molecules.

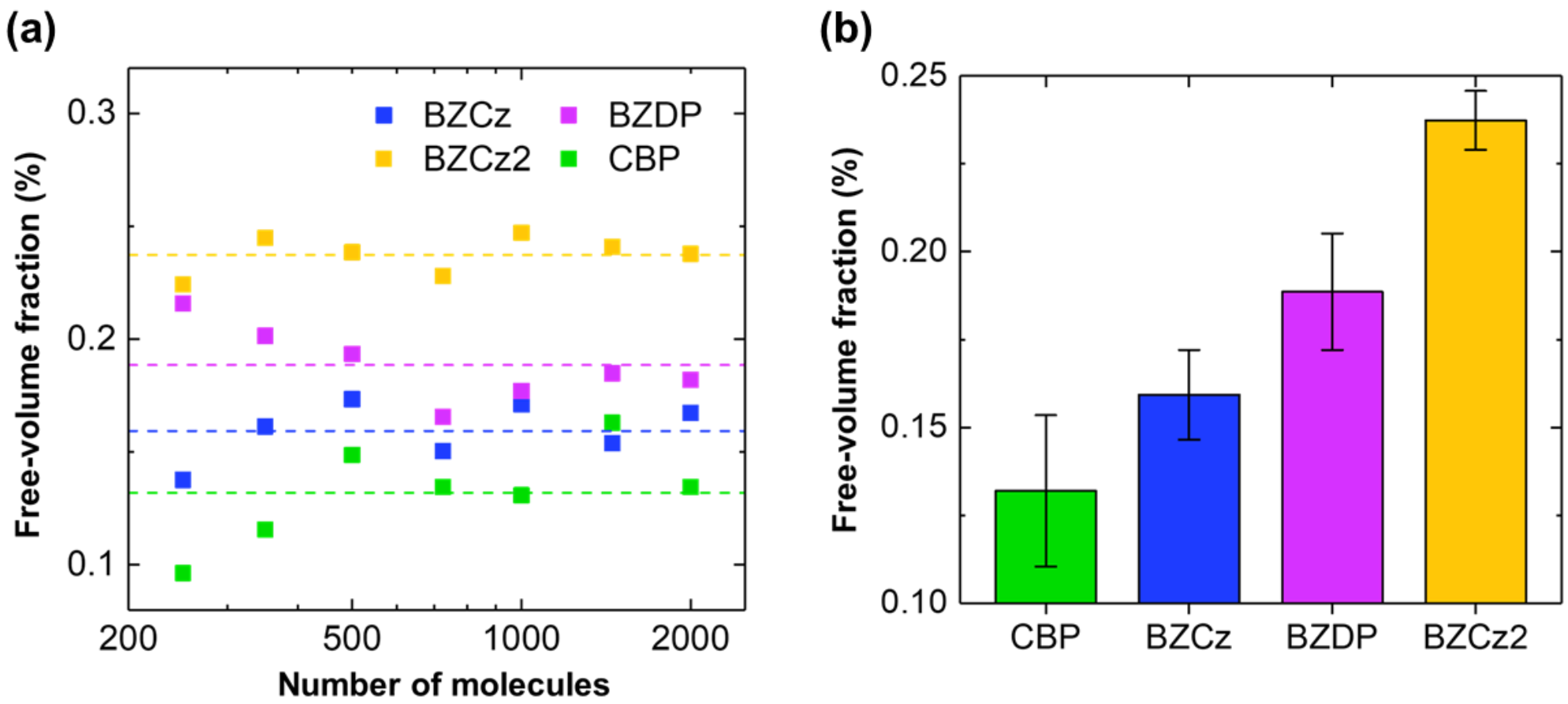

Figure S4. (a) Free-volume fractions of BZCz, BZCz2, BZDP, and CBP calculated for systems containing different numbers of molecules. Dashed lines indicate the average free-volume fraction for each material. (b) Average free-volume fractions. Error bars represent the standard deviation.

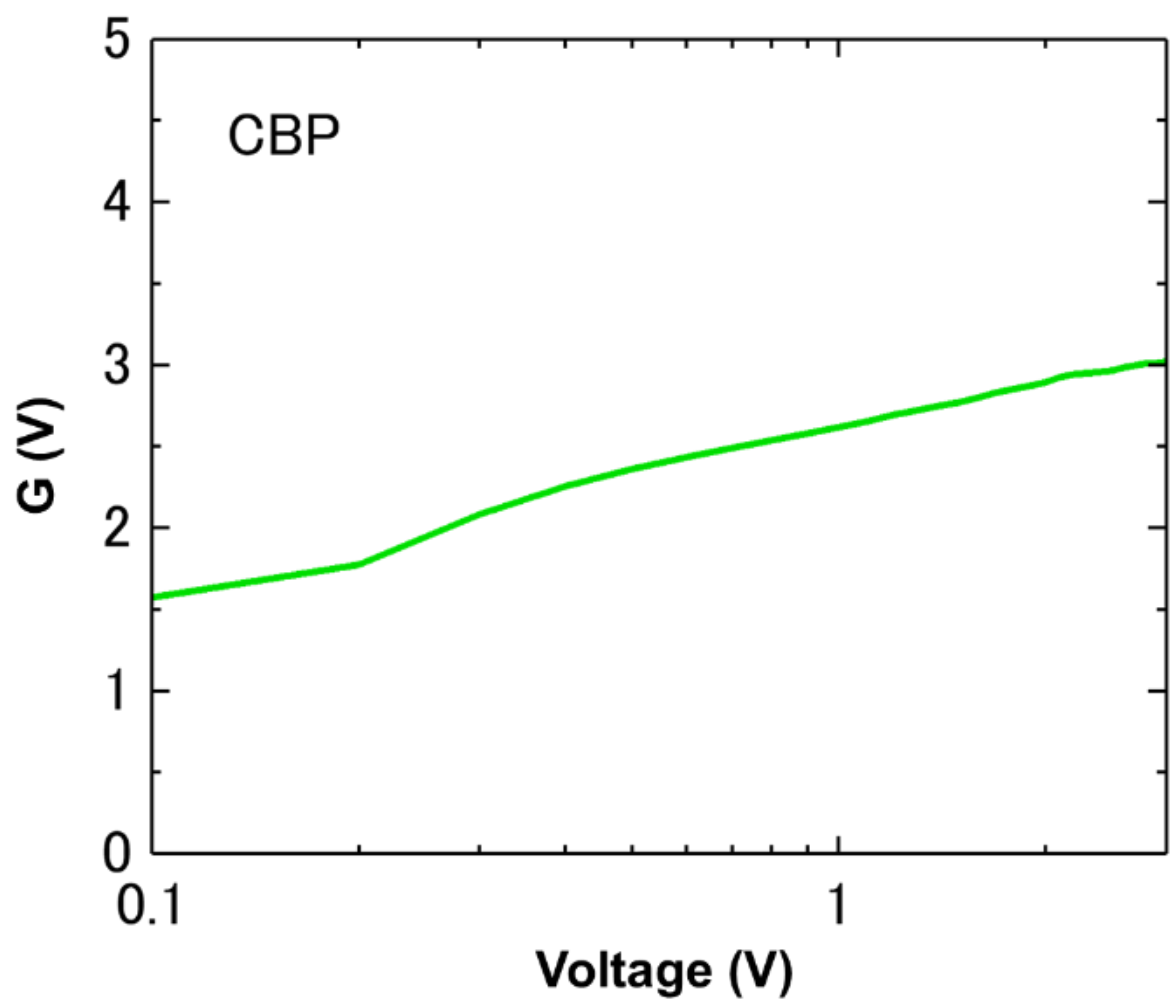

Figure S5. *G*(V)–*V* characteristics of a CBP HOD. No distinct peak was observed, and therefore V_{bi} was set to 0 V.

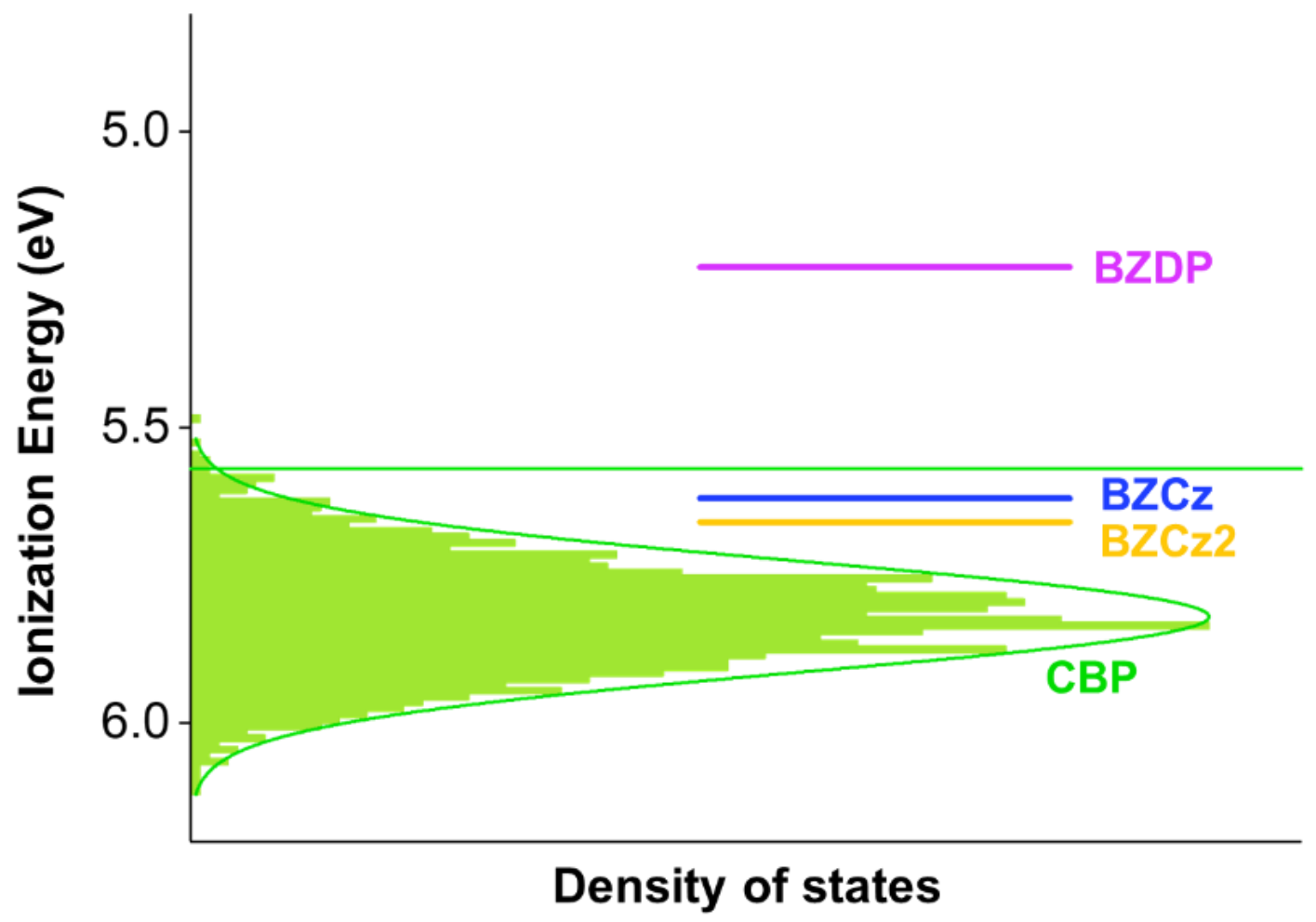

Figure S6. The solid-state distribution of DOS of MD-simulated amorphous CBP film is shown as histograms, with a Gaussian fit indicated by the green solid curve. The green horizontal line marks the onset energy of CBP, defined as an offset from the mean of the distribution by $\sigma^2/2kT$. The ionization energy levels of isolated DFT-optimized BZ derivatives are indicated by the corresponding horizontal lines.

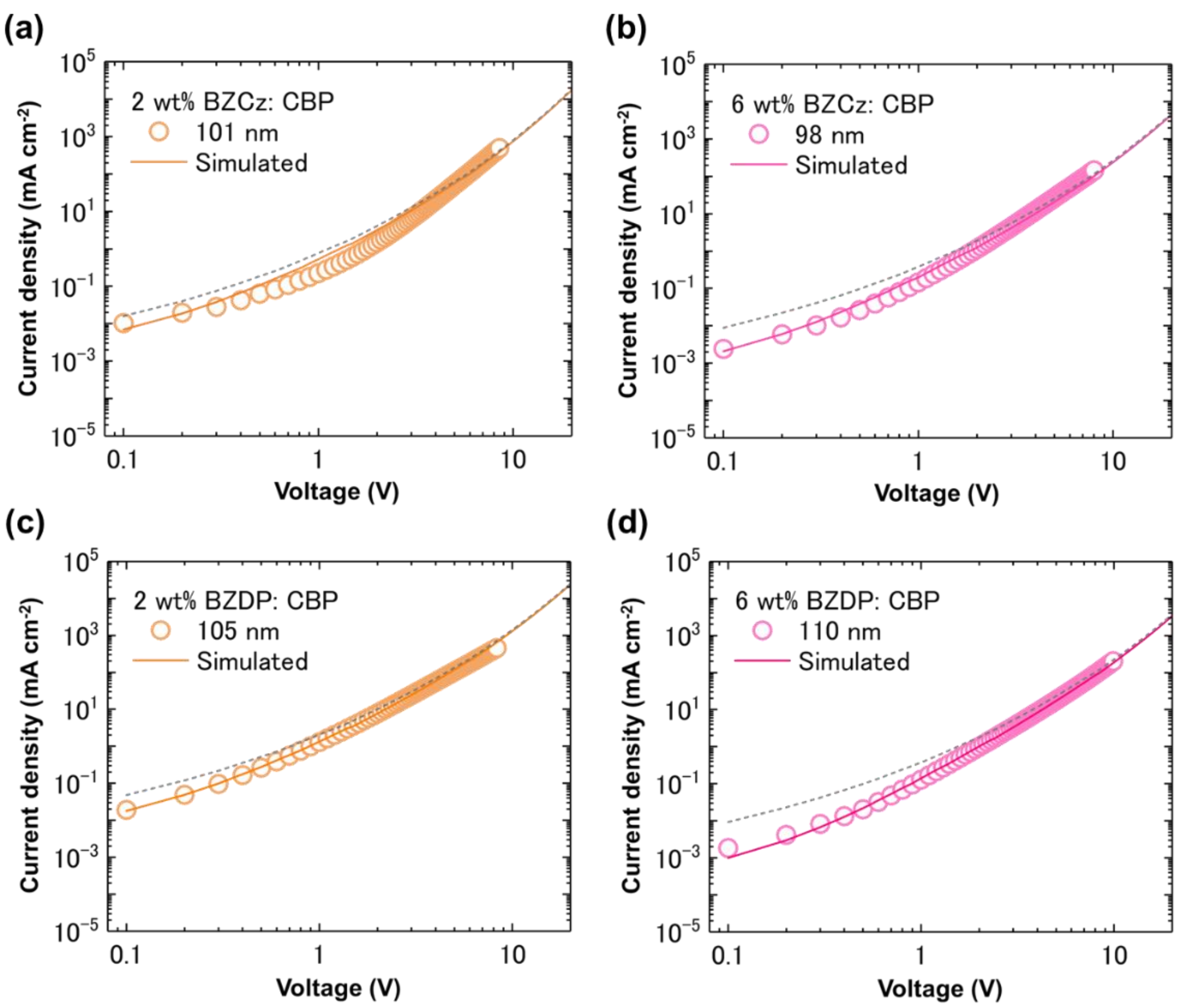

Figure S7. Experimental (symbols), simulated (solid lines), and trap-free simulated (gray dashed lines) *J*–*V* characteristics of host–guest HODs: (a) 2 wt% BZCz, (b) 6 wt% BZCz, (c) 2 wt% BZDP, and (d) 6 wt% BZDP in the CBP host.

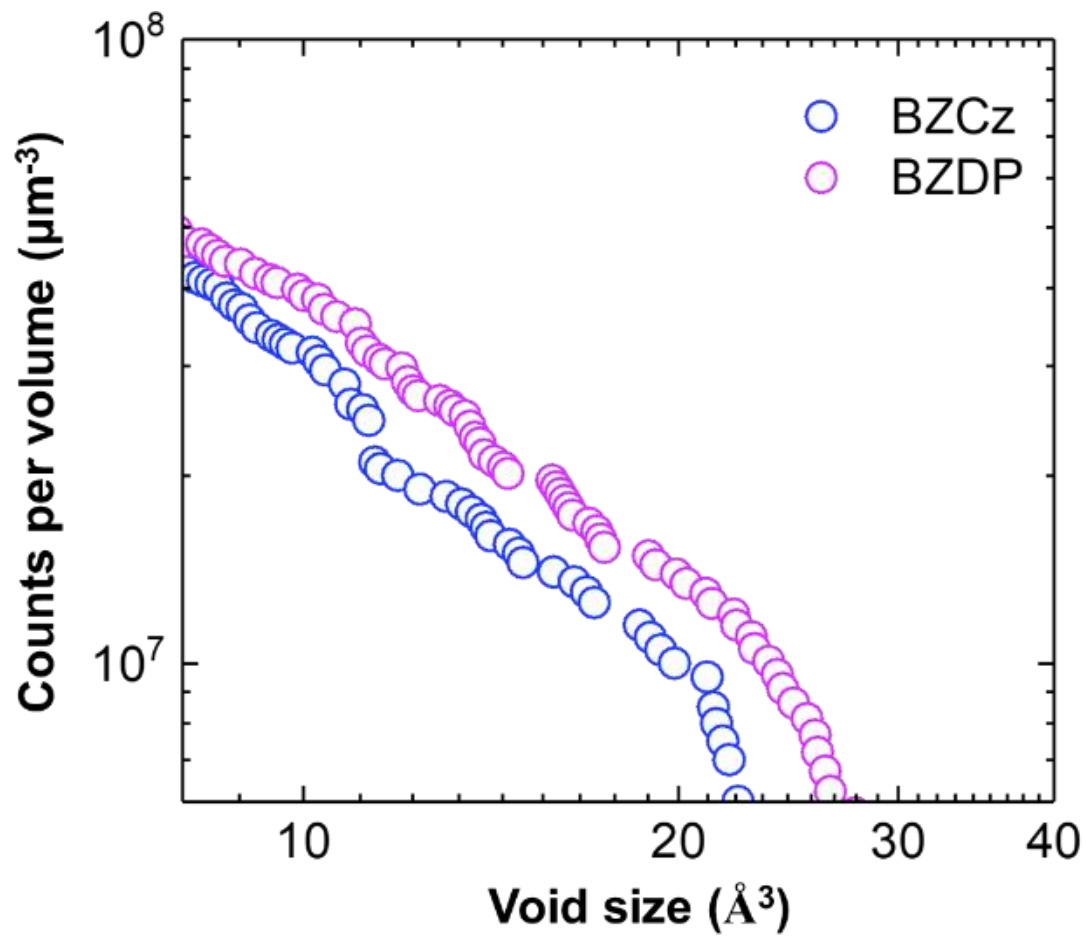

Figure S8. Cumulative void number density as a function of void size for the 2000-molecule systems of BZCz and BZDP.

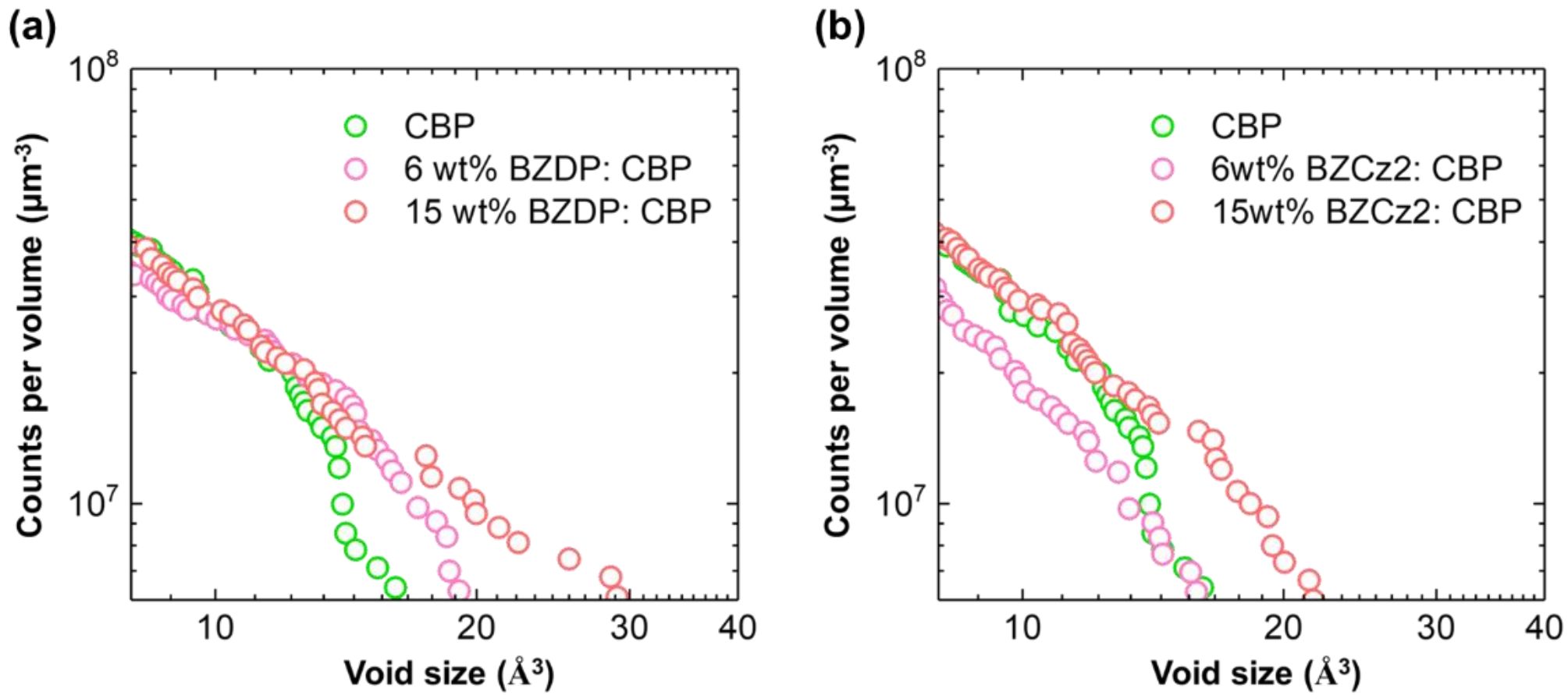

Figure S9. Cumulative void number density as a function of void size for (a) CBP, 6 wt% BZDP: CBP, and 15 wt% BZDP: CBP, and (b) CBP, 6 wt% BZCz2: CBP, and 15 wt% BZCz2: CBP systems.

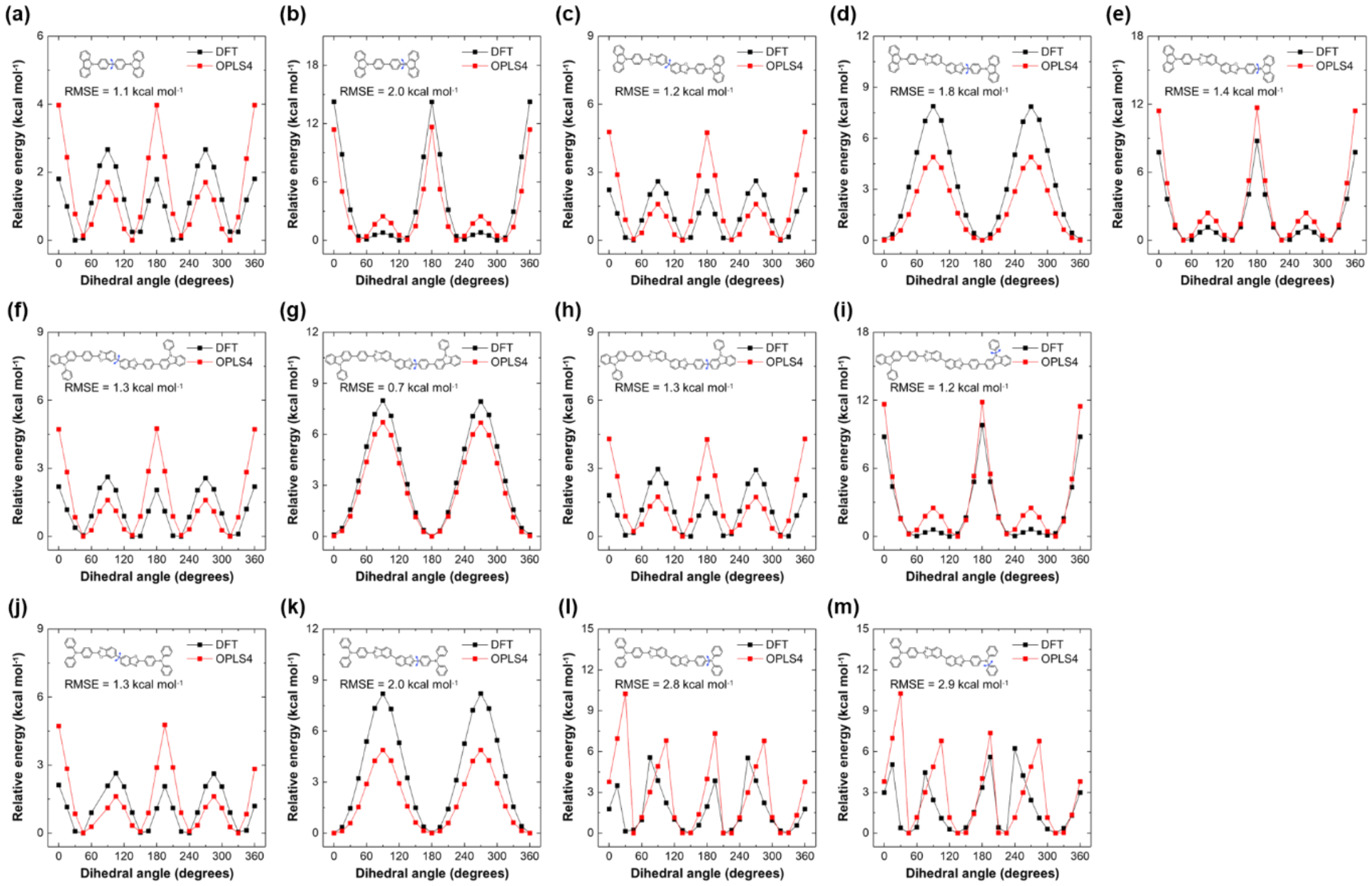

Figure S10. Comparison of torsional potential-energy profiles calculated using OPLS4 and DFT at the B3LYP/6-31G** level for representative rotatable bonds in (a–b) CBP, (c–e) BZCz, (f–i) BZCz2, and (j–m) BZDP. The relative energies for each profile were referenced to the respective minimum-energy structure for each method.

Computational details for DOS calculation

The density-of-states (DOS) calculations were performed using the VOTCA2026 package within an atomistically resolved polarizable embedding framework.[2-6] Molecular configurations were obtained from the MD simulations, and 2000 individual molecular sites were used for the DOS analysis. To account explicitly for conformational disorder, the vertical ionization energy of each molecule was evaluated at its instantaneous MD geometry. The molecular geometries were kept fixed, and single-point calculations were performed for the neutral and cationic states at the B3LYP/6-31G** level using ORCA 6.1.1.[7] The neutral and cationic states were treated with charge/multiplicity combinations of 0/1 and +1/2, respectively. The isolated-molecule vertical ionization energy at each site, i, was obtained as

$$IE_{\mathrm{vert},i} = E_i^{+} - E_i^{0}.$$

The effect of the surrounding molecular environment was subsequently evaluated using VOTCA-XTP. The environmental contribution to the site energy was separated into electrostatic, E_{stat}, and induction, E_{indu}, components. Permanent electrostatic interactions were

represented using atom-centered distributed multipoles for the neutral and cationic states, obtained by distributed multipole analysis (DMA),[8] while the induction contribution was evaluated using a polarizable force field based on the Thole model.[9] The atomic polarizabilities were rescaled to reproduce the molecular static polarizability obtained from quantum-chemical calculations. The environmental contribution was evaluated within atomistic embedding regions defined around each target molecule. The convergence of the calculated environmental correction with respect to the embedding cutoff radius was explicitly examined prior to the production calculations. Based on this convergence test, cutoff radii of 3.0 nm were adopted for both the polarizable and static regions for the calculation of the DOS. For each molecular site, the solid-state correction to the vertical ionization energy was calculated from the difference between the cationic and neutral environmental energies,

$$\Delta E_{\mathrm{stat},i} = E^{+}_{\mathrm{stat},i} - E^{0}_{\mathrm{stat},i}$$

and

$$\Delta E_{\mathrm{indu},i} = E^{+}_{\mathrm{indu},i} - E^{0}_{\mathrm{indu},i}.$$

The solid-state ionization energy of each molecular site was therefore evaluated as

$$IE_{\mathrm{bulk},i} = IE_{\mathrm{vert},i} + \Delta E_{\mathrm{stat},i} + \Delta E_{\mathrm{indu},i}.$$

The resulting set of 2000 solid-state ionization energies was used to construct the DOS distribution. In this procedure, variations in the molecular conformation contribute to the isolated-molecule ionization-energy disorder, whereas variations in the local electrostatic and polarization environments contribute additional energetic disorder.

The solid-state ionization energies of BZ derivatives were approximated using single-molecule DFT calculations in an implicit solvent with a dielectric constant corresponding to the host material, CBP. Here this was estimated to $\varepsilon_{\mathrm{r}} = 2.7$.[10] Single-point calculations were performed for the neutral and cationic states at the B3LYP/6-31G** level using the same molecular geometry for both charge states. The guest ionization energy was determined from the difference between the solution-phase total energies of the cationic and neutral states,

$$IE_{\mathrm{guest}} = E^{+}_{\mathrm{sol}} - E^{0}_{\mathrm{sol}}.$$

Reference

1. P. Mantri, S. M. H. Rizvi, and B. Mazhari, "Estimation of built-in voltage from steady-state current–voltage characteristics of organic diodes," *Org. Electron.* 14 (2013): 2034–2038.

2. V. Rühle, C. Junghans, A. Lukyanov, K. Kremer, and D. Andrienko, "Versatile object-oriented toolkit for coarse-graining applications," *J. Chem. Theory Comput.* 5 (2009): 3211–3223.
3. V. Rühle, A. Lukyanov, F. May, M. Schrader, T. Vehoff, J. Kirkpatrick, B. Baumeier, D. Andrienko, "Microscopic Simulations of Charge Transport in Disordered Organic Semiconductors," *J. Chem. Theory Comput.* 7 (2011): 3335–3345.
4. V. Rühle and C. Junghans, "Hybrid approaches to coarse-graining using the VOTCA package: Liquid hexane," *Macromol. Theory Simul.* 20 (2011): 472–477.
5. S. Y. Mashayak, M. N. Jochum, K. Koschke, N. R. Aluru, V. Rühle, and C. Junghans, "Relative entropy and optimization-driven coarse-graining methods in VOTCA," *PLoS ONE* 10 (2015): e0131754.
6. C. Scherer and D. Andrienko, "Understanding three-body contributions to coarse-grained force fields," *Phys. Chem. Chem. Phys.* 20 (2018): 22387–22394.
7. F. Neese, "The ORCA program system," *Wiley Interdiscip. Rev. Comput. Mol. Sci.* 2 (2012): 73–78.
8. A. J. Stone, "Distributed multipole analysis, or how to describe a molecular charge distribution," *Chem. Phys. Lett.* 83 (1981): 233–239.
9. B. T. Thole, "Molecular polarizabilities calculated with a modified dipole interaction," *Chem. Phys.* 59 (1981): 341–350.
10. X. de Vries, R. Coehoorn, "Vibrational mode contribution to the dielectric permittivity of disordered small-molecule organic semiconductors," *Phys. Rev. Mater.* 4 (2020): 085602.
